# Effects of the continuous use of organic manure and chemical fertilizer on soil inorganic phosphorus fractions in calcareous soil

**DOI:** 10.1038/s41598-017-01232-2

**Published:** 2017-04-25

**Authors:** Ke Song, Yong Xue, Xianqing Zheng, Weiguang Lv, Hongxia Qiao, Qin Qin, Jianjun Yang

**Affiliations:** 1 Institute of Eco-Environment and Plant Protection, Shanghai Academy of Agricultural Sciences, Shanghai, 201403 China; 2 Shanghai Scientific Observation and Experimental Station for Agricultural Environment and Land Conservation, Shanghai, 201403 China

## Abstract

A 4-year field trial with three treatments and three types of annually rotated vegetables was conducted in calcareous soil in a greenhouse using a phosphorus (P) fractionation method based on the inorganic P fraction classification system described by Jiang-Gu. With the same nutrient input, vegetable yields and P uptake were more stable under the chemical fertilizer (CF) treatment than under the organic manure (OM) treatment, and the average utilization rate of P fertilizer (URP) values were 5.27% and 11.40% under the OM and CF treatments, respectively, over the 4 years. Compared with the values in 2009, the values of the inorganic P (Pi) fractionation, including Ca-P, Al-P and Fe-P, significantly increased over time by 310.89 mg·kg^−1^, 36.21 mg·kg^−1^, and 18.77 mg·kg^−1^, respectively, with OM treatment and by 86.92 mg·kg^−1^, 175.87 mg·kg^−1^, and 24.27 mg·kg^−1^ with CF treatment. These results suggest that 1) large amounts of P were released from Ca_2_-P, Ca_8_-P and Al-P and were taken up by vegetables in the calcareous soil, and 2) the excessive application of P fertilizer, especially OM, resulted in a substantial accumulation of Pi (Ca_2_-P, Ca_8_-P and Al-P), which increased the risk of pollution from organic farming diffusing into the surface water.

## Introduction

In China, the growing prices of chemical fertilizers (CFs) and the concerns about soil quality have triggered an interest in applying organic manure (OM) to restore fertility. Moreover, the use of CFs is prohibited in organic farming; thus, OM serves as an important resource not only to supply nutrients for plants but also to replenish the organic matter content of most cultivated soils. Evidence from many studies in similar fields worldwide^1, 2^ clearly indicates the advantages of OM, which include reducing the use of CF, preventing non-point source pollution, improving soil quality and reducing the environment load. However, the disadvantages of OM compared with CF are also obvious, including lower fertilizer efficiency, lower crop yields and an imbalance of nitrogen (N), phosphate and potassium fertilizer (NPK). On average, the proportion of NPK content in OM is not suitable for plant growth, which results in N deficiency but phosphorus (P) accumulation.

Due to a slower nutrient release and the out-of-proportion NPK content, crop yields are lower under OM farming than under CF farming. To achieve high yields and a high economic output, the heavy dressing of OM has been adopted in China. As a result, excess fertilization under OM farming is frequently observed, especially in vegetable greenhouses. In many areas, the excessive application of fertilizer and manure has increased soil P levels^3, 4^. It has also been established that only one-quarter to one-third of the P fertilizer is utilized, while large amounts of P accumulate in the soil^5^. Excessive P can lead to plant toxicity and the immobilization of trace metals in the soil, as well as ground and surface water pollution through runoff.

Compared with other nutrients, P is the least mobile and least available for plants in soil, especially in calcareous soil. Therefore, P was greatly affected by fertilization. P fractionation schemes have been used to evaluate the effects and relationship between the P fraction and the P that is available for plants. Therefore, it is important to understand the forms and characteristics of P in soil for developing long-term management strategies. The method developed by Chang and Jackson^6^ and Buehler *et al*.^7^ has been widely used to estimate the soil P fractionation in non-calcareous soil. Soil P in non-calcareous soil was divided into different fractions, including Ca-P, Al-P, Fe-P and occluded P (O-P); in calcareous soil, Ca-P was further divided into Ca_2_-P, Ca_8_-P and Ca_10_-P according to the solubility and availability of P^8, 9^ because the Ca-P content was relatively higher in calcareous than in non-calcareous soil.

The effects of CF or OM on P fractionation, inorganic P (Pi) transformation in soil and Pi availability and recovery by crops have been investigated in several studies. These studies have reported the accumulation of a large amount of Pi or organic P in the soil due to the continuous application of P fertilizer or OM for many years^10–12^, but most studies on calcareous soils have been related to wheat, rice or maize^13, 14^. There have been few reports on soil P fractionation and Pi transformation in response to the fertilization of vegetables, especially greenhouse vegetables; therefore, the purpose of the current study was as follows: 1) to investigate the response of vegetable yields to different fertilization inputs, such as OM and CF; 2) to evaluate P availability with different fertilization inputs; and 3) to study the transformation of Pi fractions in calcareous soil under excess fertilizer application and vegetable uptake in the greenhouse.

## Materials and Methods

### Study site

The study site was located at the Dahong vegetable experimental station of Shanghai Academy of Agriculture Sciences, Pudong New District (31°11′27″N, 121°42′20″E), in eastern Shanghai, China. This area has a subtropical monsoon climate and an ocean coastline with an annual average precipitation of 1100 mm and an annual average temperature of 15.8 °C. The experimental station covers an area of 4.5 hm^2^. Vegetables have been cultivated for more than 10 years, and the majority are planted in greenhouses. The topsoil (0–30 cm) at the research site is vegetable garden soil and evolved from silt loam.

### Experimental design

A comparative greenhouse experiment was conducted from 2009–2013 to examine vegetable yields, P fractionation and Pi availability in response to different fertilizer inputs to calcareous soil. Two fertilization modes with one CK treatment were included in the experiment: (1) OM, (2) CF, and (3) no fertilizer (CK). According to the organic cultivation mode in China, only OM was applied in the OM treatment, while only CF was used in the CF treatment. No fertilizer was applied on the CK plots during the period of the experiment. The three treatments were applied in one greenhouse, which was newly built and based on an open vegetable field in the experiment station. The topsoil contained organic matter, 22.67 g·kg^−1^; bulk density, 1.32 g·kg^−1^; total N, 1.85 g·kg^−1^; alkali-hydrolysable N, 76.08 mg·kg^−1^; available P, 55.33 mg·kg^−1^; and available K, 148.34 mg·kg^−1^, and the pH was 7.46 (water:soil ratio = 5:1). Each experimental treatment had three plots with an area of 30 × 2 = 60 m^2^ each, and the same farming practices were applied in each treatment.

### Experimental procedure

Three types of vegetables were rotated successively each year of the experiment: carrot (Zheluo No. 1), cucumber (Hu No. 58) and purple cabbage (Xiaguang); all vegetables were provided by the Shanghai Horticulture Research Institute, China. Plantings were conducted four times for each type of vegetable from 2009–2013. Carrot was planted from March to June, cucumber was planted from July to November, and purple cabbage was planted from December to February. Vegetable cultivation and management were synchronous in all treatment plots. There was no fertilization in the CK treatment. OM was applied at 30,000 kg·hm^−2^ for cucumber and 15,000 kg·hm^−2^ for purple cabbage and carrot each year as a basal fertilizer in the OM treatment; only one type of OM was used during the experimental period, and it contained N at 11.5 mg·kg^−1^, P_2_O_5_ at 20.4 mg·kg^−1^, and K_2_O at 15.1 mg·kg^−1^. However, NPK was added in the form of urea, calcium superphosphate and potassium chloride at the rate of 46% N, 16% P_2_O_5_ and 50% K_2_O in the CF treatment. P and K fertilizers were all applied as basal fertilizers for each type of vegetable along with 50% N as the basal fertilizer and 50% as the top-dressing. The rate of each type of CF was determined according to the level of pure nutrients from the OM that was applied in the OM treatment (Table 1). Except for fertilization, all other aspects were equal in the different treatments.

**Table 1:** Varieties and amounts of fertilizer each year under the different treatments.

Treatments	Fertilizer		Pure nutrient (kg·hm^−2^)		
	Varieties	Amount (kg·hm^−2^)	N	P_2_O_5_	K_2_O
OM	Organic manure	60,000	930	1224	906
CF	urea	2021.74	930	0	0
	calcium superphosphate	7650	0	1224	0
	potassium chloride	1812	0	0	906
CK	\	\	0	0	0

### Soil sampling and analysis

The initial soil sample was a composite consisting of 5 soil cores, which were collected in an “S” pattern from the 0–20 cm topsoil layer in each greenhouse with a soil corer (5 cm in diameter) after the rice harvest, and after vegetable cultivation, soil samples were collected annually in the same way in November after the carrot harvest. Each composite sample was mixed and air dried for 10 days after the crop roots and pebbles were discarded. All the samples were passed through 2-mm and 0.149-mm sieves to analyse the various indicators.

The soil organic matter was measured by wet oxidation. The soil pH was measured with a glass electrode after mixing the soil with water (1:5 wt/vol soil:water), and the soil bulk density was determined by pushing a 100 cm^3^ stainless steel cylinder into the soil vertically and oven drying the soil samples to a constant weight at 105 ± 2 °C (normally within 24 h). The total N was determined by a micro-Kjeldahl digestion method and the total P was digested with H_2_SO_4_ and HClO_4_ and measured using molybdenum blue spectrophotometry. Alkali-hydrolysable N was measured using an alkali solution diffusion method; available P was measured via the Olsen-P method; and the available K was determined using atomic absorption spectrophotometry^15^.

The P fractionation method was based on the Pi fraction classification systems described by Jiang and Gu^16^. Six Pi fractions (Ca_2_-P, Ca_8_-P, Al-P, Fe-P, O-P and Ca_10_-P) were extracted from the soil samples using a sequential extraction procedure. Ca_2_-P was removed using a sodium bicarbonate solution (c (NaHCO_3_) = 0.25 mol/L, pH 7.5); (2) Ca_8_-P was removed using an ammonium acetate solution (c (CH_3_COONH_4_) = 0.5 mol/L, pH 4.2); (3) Al-P was removed using an ammonium fluoride solution (c (NH_4_F) = 0.5 mol/L, pH 8.2); (4) Fe-P was removed using 0.1 M NaOH and 0.1 M Na_2_CO_3_; (5) O-P was removed using a sodium citrate solution (c (Na_3_C_6_H_5_O_7_·2H_2_O) = 0.3 mol/L); and (6) Ca_10_-P was removed using a sulphuric acid solution (c (1/2H_2_SO_4_) = 0.5 mol/L).

### Plant sampling and analysis

The vegetables were harvested after physiological maturity, and the yields were recorded. The plant samples were collected, dried and digested with H_2_SO_4_ and H_2_O_2_, and the P concentration in the digest was determined colourimetrically using the vanado-molybdate- yellow colour method^17^. The dry-matter yields and P concentration were used to calculate the total P uptake by crops, the utilization rate of P fertilizer (URP) and the agronomic efficiency of P (AEP).

The URP under the OM and CF treatments was calculated as follows:

1 $${\rm{URP}}=100\ast \frac{{{\rm{P}}}_{{\rm{t}}}-{{\rm{P}}}_{0}}{{{\rm{P}}}_{{\rm{f}}}}$$

where URP is the utilization rate of P fertilizer; P_t_ is the P uptake under the OM or CF treatment; P_0_ is the P uptake under the CK treatment; and P_f_ is the amount of P fertilizer applied.

The AEP under the OM and CF treatments was calculated as follows:

2 $${\rm{AEP}}=\frac{{{\rm{Y}}}_{{\rm{t}}}-{{\rm{Y}}}_{0}}{{{\rm{P}}}_{{\rm{f}}}}$$

where AEP is the agronomic efficiency of P, Y_t_ is the crop yield under the OM or CF treatment; Y_0_ is the crop yield under the CK treatment; and P_f_ is the amount of P fertilizer applied.

### Statistical analysis

All the experiments were repeated in triplicate, and the mean value was accepted as the final value. The data were processed using Microsoft Excel 2010 (Microsoft Corp., Redmond, WA, USA) and then subjected to analysis of variance (ANOVA) using the SPSS 17.0 Statistical Package (SPSS Inc., Chicago, IL, USA). Multiple comparisons of the various treatments were conducted using Tukey’s method. The graphics were produced using Origin 8.0 (OriginLab Corp., Northampton, MA, USA).

## Results

### Vegetable yields in response to fertilization

**Figure 1:**
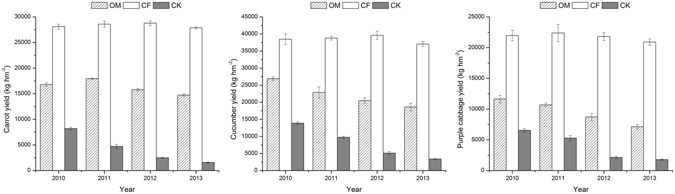
Changes in the different types of vegetable yields under the different fertilizer treatments during the 4 years of cultivation.

Different fertilization modes led to regular changes in vegetable yields under the different treatments (Fig. 1). For the CF treatment, the vegetable yields remained at a relatively stable level for the first three years but began to decrease for purple cabbage in 2011 and for cucumber and carrot in 2012. Under the OM treatment, the yields of any one type of vegetable in 2013 were dramatically lower than that in 2010. However, among the 4 years, the trend of the yields for purple cabbage and cucumber decreased, while carrot yields were the highest in 2011 and gradually decreased later. Under CK treatment, the yields of all three types of vegetables rapidly decreased with increasing time during the experiment, but the decreasing trend slowed. There was a significant difference in the yields of vegetables among the three treatments, and the yields of vegetables under the CF and OM treatments were significantly higher than under the CK treatment. After four years of cultivation, the average yields of carrot, cucumber and purple cabbage under the CF treatment improved by 89%, 50% and 193%, respectively, compared with those under the OM treatment.

### Change in the total P and Olsen-P

Table 2 shows the change in Olsen-P and total P affected by different fertilization modes in the surface soil layer (0–20 cm) over time. Olsen-P had a similar concentration value, and there was no significant divergence among the treatments in 2009 before the experiment; however, the different fertilization modes strongly affected Olsen-P accumulation in the 0–20 cm soil layer. Compared with the value in 2009, the concentration of Olsen-P increased by 56.56% and 30.85% under OM and CF treatments, respectively, in 2013. The data indicated a marked accumulation of Olsen-P in the soil with the current P fertilizer amount, under both the OM and the CF treatments. In contrast, the concentration of Olson-P decreased under the CK treatment, which was 64.31% lower in 2013 than in 2009. This trend was due to the plant absorption of P in soil without an application of P fertilizer. Therefore, in the comparison of the different treatments, there was a significant difference between OM or CF and CK from 2011 and between OM and CF from 2012.

**Table 2:** Change in total P and Olsen-P affected by different fertilization modes in the surface soil layer (0–20 cm) over time.

Items	Treatments	Soil P content (mg·kg^−1^) over time (years)				
		2009	2010	2011	2012	2013
Olsen-P	OM	54.96 ± 7.07aA	57.35 ± 9.85aA	62.50 ± 15.28aA	72.74 ± 15.46aA	86.04 ± 14.53aA
	CF	55.33 ± 11.41aA	56.44 ± 10.34aA	58.42 ± 13.05aA	65.58 ± 15.10bA	72.40 ± 10.16bB
	CK	54.72 ± 8.58aA	51.23 ± 6.42aA	46.22 ± 10.46bB	34.09 ± 10.44cB	19.53 ± 7.42cC
Total P	OM	1327.36 ± 86.45aA	1326.49 ± 72.48aA	1358.36 ± 82.51aA	1402.47 ± 77.52aA	1488.04 ± 105.27aA
	CF	1332.46 ± 90.37aA	1325.37 ± 117.33aA	1348.32 ± 134.26bA	1362.41 ± 154.62bB	1392.56 ± 152.30bB
	CK	1321.27 ± 45.28aA	1320.48 ± 50.42aA	1321.32 ± 62.37cB	1319.46 ± 66.48cC	1317.70 ± 72.35cC

Note: The different lowercase and uppercase letters in the same column for each parameter indicate a significant difference at the 0.05 and 0.01 levels, respectively.

Soil total P concentrations at the 0–20 cm depth increased under the OM and CF treatments by 12.11% and 4.51%, respectively, but decreased under the CK treatment over 4 years of cultivation. There was a significant difference (*P* < 0.05) among the OM, CF and CK treatments from 2011 and (*P* < 0.01) 2012. In addition, the soil total P concentrations under OM and CF were 12.93% and 5.68% higher, respectively, than those under CK in 2013.

### Partial balance of P

As shown in Table 3, P input mainly occurred from fertilization, and other sources of P, including rainfall and irrigation, were neglected. Additionally, P output was only evaluated as plant uptake without considering the small leaching losses in the greenhouse, so the P input was far greater than its output under both treatments. The partial P balance budget indicated a net gain of approximately 491.26 kg·hm^−2^ and 458.52 kg·hm^−2^ per year under the OM and CF treatments, respectively, over the 4 years of fertilization. The amount of plant P uptake was greater under CF than OM because the vegetable yields were much higher under CF (Fig. 1). However, there was an obvious depletion of P under the CK treatment, and the amount of plant P uptake was much lower than in the fertilized control. Under the OM and CF treatments, the average URP values were 5.27% and 11.40% for 4 years, respectively. The URP is often low and rarely exceeds 20% for P in agrosystems^18^. This condition was also observed in the present study, but the value of OM was much less than that of CF. The same trend appeared for AEP, and the mean under OM and CF was 59.63 kg·hm^−2^ and 135.40 kg·hm^−2^, respectively.

**Table 3:** The P balance, URP and AEP values under the different treatments.

Year	Treatments	P input (kg·hm^−2^)	Plant P uptake (kg·hm^−2^)	P balance (kg·hm^−2^)	URP (%)	AEP (kg·hm^−2^)
2010	OM	534.42	51.00 ± 6.57bB	483.42 ± 38.42aA	4.64 ± 1.45bB	50.06 ± 6.48bB
	CF	534.42	75.83 ± 5.42aA	458.59 ± 42.56bA	9.29 ± 3.26aA	112.15 ± 13.24aA
	CK	0	26.18 ± 3.18cC	−26.18 ± 5.44cB	—	—
2011	OM	534.42	45.12 ± 3.67bB	489.30 ± 41.68aA	5.06 ± 1.87bB	59.42 ± 5.73bB
	CF	534.42	76.64 ± 12.15aA	457.78 ± 46.75bB	10.95 ± 4.21aA	131.04 ± 15.25aA
	CK	0	18.10 ± 1.14cC	−18.10 ± 3.54cC	—	—
2012	OM	534.42	40.10 ± 4.55bB	494.32 ± 58.47aA	5.75 ± 2.14bB	65.95 ± 6.14bB
	CF	534.42	77.80 ± 6.89aA	456.62 ± 32.48bB	12.81 ± 4.35aA	150.47 ± 16.28aA
	CK	0	9.36 ± 1.42cC	−9.36 ± 2.56cC	—	—
2013	OM	534.42	36.42 ± 5.47bB	498.00 ± 62.14aA	5.64 ± 3.37bB	63.07 ± 4.79bB
	CF	534.42	73.34 ± 6.64aA	461.08 ± 35.41bB	12.55 ± 4.56aA	147.95 ± 15.33aA
	CK	0	6.26 ± 0.89cC	−6.26 ± 3.42cC	—	—

Note: The different lowercase and uppercase letters in the same column for each year indicate a significant difference at the 0.05 and 0.01 levels, respectively.

### Soil inorganic phosphorus fractions

Soil Pi fractionation was conducted on the calcareous soils^8, 15^, and the results are listed in Table 4. The Pi concentration was exactly same among the treatments in 2009, and the dominant Pi fraction was O-P (46.08–48.99%), followed by Fe-P (17.54–19.37%), Ca-P (17.04–18.58%) and Al-P (15.17–17.11%). For Ca-P, the fractions of Ca_2_-P accounted for 31.25–34.21%, Ca_8_-P for 36.73–39.90%, and Ca_10_-P for 28.85–29.91%. In addition, the order of the 6 fractions by concentration was O-P > Al-P > Fe-P > Ca_8_-P > Ca_2_-P > Ca_10_-P.

**Table 4:** Changes in the concentration of inorganic Pi in the surface soil layer (0–20 cm) as affected by different fertilization modes (mg·kg^−1^).

Time	Fertilization mode	Ca_2_-P	Ca_8_-P	Al-P	Fe-P	O-P	Ca_10_-P	Sum of the fractions
2009	OM	55.84 ± 8.34aA	68.42 ± 12.46aA	162.24 ± 25.57aA	183.43 ± 18.33aA	472.46 ± 38.42aA	51.65 ± 6.28aA	994.04aA
	CF	56.42 ± 10.57aA	67.75 ± 12.58aA	161.53 ± 32.14aA	178.16 ± 23.49aA	467.56 ± 41.64aA	51.86 ± 7.46aA	983.28aA
	CK	58.06 ± 5.10aA	65.01 ± 7.35aA	157.9 ± 18.62aA	177.24 ± 15.24aA	468.65 ± 32.16aA	51.43 ± 3.15aA	978.29aA
2013	OM	140.99 ± 21.38aA	293.36 ± 35.24aA	196.77 ± 36.22bB	198.38 ± 21.48aA	475.01 ± 42.10bAB	52.02 ± 15.34aA	1356.53aA
	CF	102.4 ± 20.84bB	107.25 ± 18.47bB	333.77 ± 75.41aA	201.51 ± 28.36aA	521.87 ± 51.27aA	51.77 ± 17.28aA	1318.57aA
	CK	16.37 ± 2.41cC	31.93 ± 9.37cC	127.15 ± 20.54cC	178.96 ± 15.47bA	455.88 ± 23.10bB	51.49 ± 8.47aA	861.78bB

Note: The different lowercase and uppercase letters in the same column for each year indicate a significant difference at the 0.05 and 0.01 levels, respectively.

A significant change occurred for inorganic P fractions under the treatments with different fertilization modes after the course of the four-year experiment. The concentrations of Ca-P, Al-P and Fe-P significantly increased under both OM and CF treatments by 310.89 mg·kg^−1^, 36.21 mg·kg^−1^, and 18.77 mg·kg^−1^ in 2013 compared with 2009 under the OM treatment and 86.92 mg·kg^−1^, 175.87 mg·kg^−1^, and 24.27 mg·kg^−1^ under the CF treatment, respectively. Significant differences were observed in the concentrations of Ca_2_-P, Ca_8_-P, and Al-P (*P* < 0.01) between the OM and CF treatments. The concentrations of Ca_2_-P and Ca_8_-P under the OM treatment were significantly higher than those under the CF, whereas Al-P and Fe-P were lower in the OM. In contrast, the concentrations of Ca_2_-P, Ca_8_-P and Al-P decreased under the CK treatment with values significantly lower (*P* < 0.01) than those under both the OM and CF treatments in 2013. However, Fe-P, O-P and Ca_10_-P remained at a relatively stable level.

With the increase in P, the total Pi increased significantly under both the OM and CF treatments due to gains of 491.26 kg·hm^−2^ (average value) and 458.52 kg·hm^−2^ per year, respectively. In contrast, the reduced partial P balance explained the depletion of total Pi under the CK treatment.

## Discussion

The difference in the vegetable yields responding to fertilization under the different treatments emphasized the importance of studying the relationship between vegetable yields and soil nutrient supply with different fertilization modes in the vegetable greenhouse^16^. Fertilizer application clearly increased the yield compared with that of the control, and CF application significantly increased the yield compared with the yields obtained under OM application. This difference could be attributed to the efficiency of OM, which was significantly lower than that of CF; this finding agrees with a previous report^18^.

The relatively stable vegetable yields under CF treatment compared with those under OM treatment also suggested a substantial nutrient supply capacity for vegetable growth. The vegetable yields under the OM treatment decreased over time, which indicated that a single application of OM could not supply enough nutrients for vegetable growth, although the soil nutrient content increased over time. Under the CK treatment, there was a significant decrease in vegetable yield due to the lack of nutrient application, which indicated that nutrient depletion constrained vegetable growth; the Olsen-P content declined from 54.72 mg·kg^−1^ in 2009 to 46.22 mg·kg^−1^ in 2011 and further decreased to 19.53 mg·kg^−1^ in 2013.

Soil available P is considered a major factor in both evaluating the soil P supply capacity and determining the P fertilization rate. In calcareous soils, the Olsen-P concentration is a good indicator for estimating soil P availability^19^. In this study, Olsen-P was referred to as ‘available P’ to emphasize its availability. The concentration of Olsen-P increased over time under the OM and CF treatments, which indicates that fertilization with OM or CF increased the Olsen-P content of the soil. The concentration of Olsen-P under the OM treatment was higher than that under the CF treatment. There might be two reasons for this difference: 1) The higher vegetable yields depleted more available P under the CF treatment (N, not P, was the critical factor for increasing vegetable yields, and N was more available under the CF treatment than under the OM treatment, which led to higher crop yields under the CK treatment); and 2) OM was able to activate soil P^20, 21^. The removal of P by crops caused the soil available P to constantly decrease under the CK treatment, in which no P was applied. This decrease may have occurred because soil Olsen-P was available for vegetable growth.

After four consecutive cropping seasons, the balance of soil P was positive under both OM and CF treatments. Moreover, P input was much higher than P output in soil because the application of P fertilizer was far greater than the plant P uptake. Less soil P was absorbed by vegetable plants under the OM treatment, which increased the P remaining in the soil, than by those under the CF treatment. Therefore, more P accumulated in the OM-treated soil. The high accumulation of P in the vegetable field not only could result in Fe, Ca, Mg and Zn becoming unavailable for vegetables but also might be one factor negatively affecting the water in the environment. The P balance under the CK treatment was negative in every growing season because there was no P fertilizer input. Excessive fertilization resulted in a low URP under both the OM and CF treatments, ranging from 4.64 to 5.75% and 9.29 to 12.81%, respectively. The URP under the CF treatment was roughly twice that under the OM treatment because the vegetable plants absorbed more P when fertilized with CF. The same trend appeared for the AEP as well, and the absorbed P that contributed to the vegetable yield can be determined effectively based on AEP.

**Figure 2:**
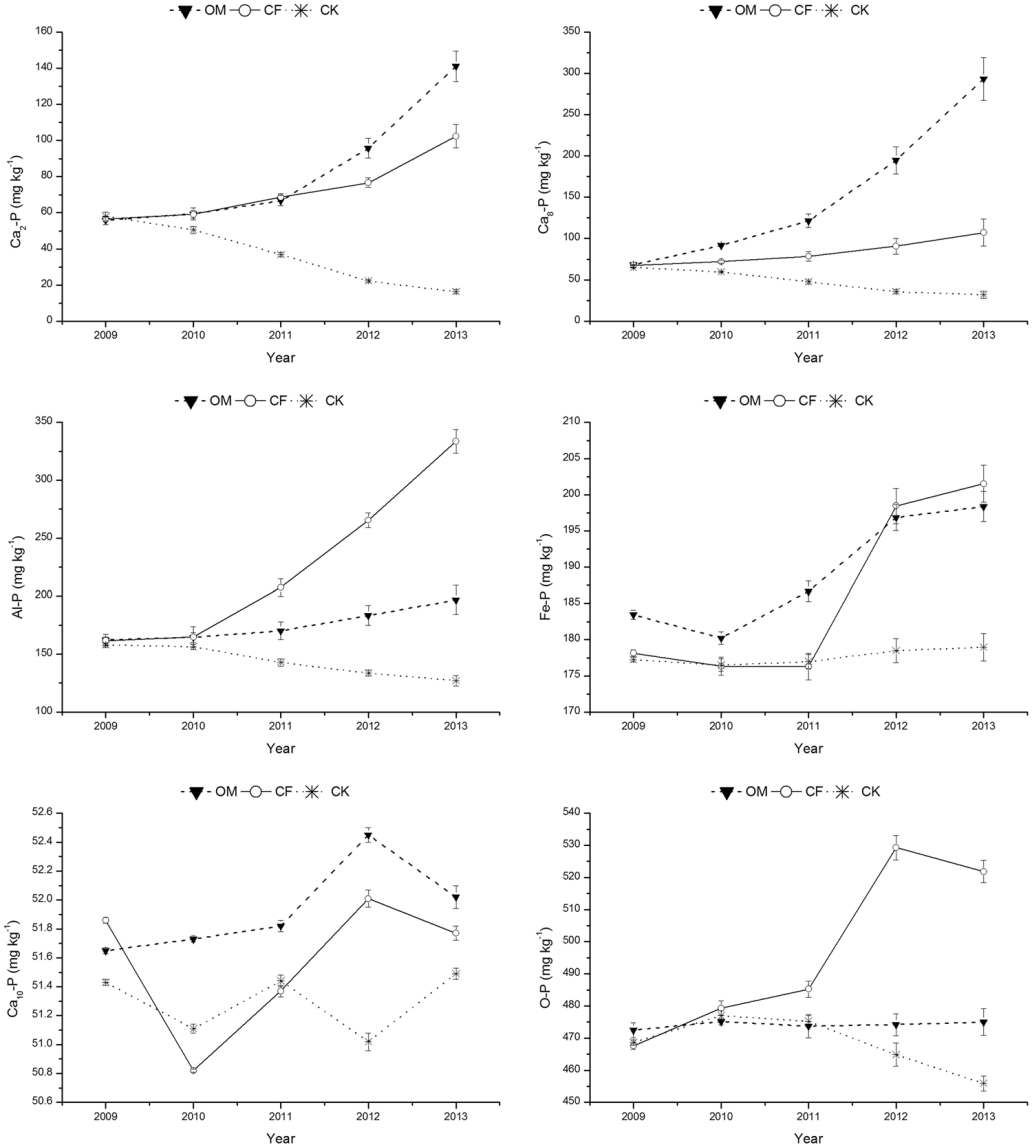
Changes in different Pi fractions over time under different treatments during the experimental period.

P exists in soil in various forms and is available to plants at different times^22^. Figure 2 presents changes in different P fractions in the soil after 4 years of cultivation in the greenhouse, and significant differences between fertilization modes were found in P fractions after the 4-year experiments (Table 5). OM treatment generally presented higher Ca_2_-P and Ca_8_-P accumulation than did CF treatment in the greenhouse, whereas for Al-P and Fe-P, the opposite trend was observed, which suggested the influence of a different fertilization mode for biologically available P. The P fractions with higher biological availability accumulated in the topsoil possibly due to the higher soil organic matter under the OM treatment^23^. The accumulation of more biologically available P was similar to the accumulation of organic residues in the topsoil. The P sorption sites could be occupied by organic matter fractions and decrease P retention^24^. Therefore, P availability could be improved. These interactions may partially explain the higher values of the available P fractions (Ca_2_-P and Ca_8_-P) observed under the OM treatment in this study. These results also indicate that OM readily incorporated P from fertilizer into the available P fraction.

**Table 5:** The correlation coefficient (*r*) between different phosphorus fractions and Olsen-P under different treatments.

Treatments	Coefficient of correlation					
	Ca_2_-P	Ca_8_-P	Al-P	Fe-P	O-P	Ca_10_-P
OM	0.991**	0.999**	0.938**	0.831*	0.522	0.642
CF	0.988**	0.995**	0.953**	0.846*	0.462	0.476
CK	0.967**	0.959**	0.842*	0.511	0.357	0.122

*P ≤ 0.05 and **P ≤ 0.01.

Neither Al-P nor Fe-P were immediately available for plants but had the potential to become available after a relatively slow process because there is a dynamic balance between available P and non-available P; thus, Al-P and Fe-P represented the moderately available P pool in the calcareous soil. However, excessive fertilization seemed to have upset the balance in the vegetable greenhouse. Thus, the contents of Al-P and Fe-P increased rapidly during the four years of the experiment under the fertilization treatments, especially under the CF treatment, which was most likely a result of decreased soil pH, which is considered an important factor for vegetable growth due to the heavy application of inorganic N fertilizer^25^.

O-P and Ca_10_-P represented non-labile or stable P compounds. The O-P and Ca_10_-P contents were relatively stable in the four-year experiment, which confirmed that the large amount of P fixed in the calcareous soil is responsible for the long-term release of P by pedological processes^26^. This observation suggested that non-labile P was almost not affected by the excessive addition of fertilizer P. However, a decline in the O-P status of the CK treatment soil was observed because extracellular phosphate enzymes released by plants and microbes facilitate the mineralization of P_0_ when the available P concentration of the soil solution is low^27^. This trend was also reported by Zhang *et al*.^28^, who found that the soil P_0_ of non-fertilized plots decreased by 14% of the initial value over 5 years of maize production.

There was a significant positive correlation (*P* ≤ 0.01) between Olsen-P and Ca_2_-P, Ca_8_-P or Al-P (*P* ≤ 0.05) and between Olsen-P and Fe-P under both the OM and CF treatments but not between O-P and Ca_10_-P (Table 5). These results reflect the relation between the different Pi fractions and Olsen-P. As available P, Olsen-P had a higher correlation coefficient with Ca_2_-P and Ca_8_-P under the OM treatment than under the CF treatment but a lower correlation with Al-P and Fe-P. This observation suggests the different potentials of P release and availability from these fractions with different fertilization modes. However, based on previous studies, there was no significant positive relationship between Olsen-P and Fe-P^29, 30^. The difference may be due to a large input and surplus of P fertilizer in the current study. Under the CK treatment, Olsen-P also had a significant (*P* ≤ 0.01) positive correlation with Ca_2_-P or Ca_8_-P (*P* ≤ 0.05) and with Al-P, which could indicate that these fractions provided available P for the vegetables in the experimental soil. Both Ca_2_-P and Ca_8_-P were readily used by plants because these Ca-P fractions released water-soluble and citrate-soluble P under the Pi fraction scheme^15^. There was no significant correlation between Olsen-P and O-P or Ca_10_-P under any of the treatments, suggesting that both P fractions had very low availability to the plants^31^.

As shown in Table 6 and compared with data from 2009, the soil organic carbon content increased under the OM treatment and decreased under the CF and CK treatments in 2013, which suggests that the application of OM is necessary to improve the soil organic carbon supply in the vegetable greenhouse. Other studies^32^ reported no clear change in organic carbon in the soil of rice systems even without fertilizer application, which is mainly due to crop residues remaining in the fields. However, this trend was not observed for vegetable cultivation. OM application also contributed to the maintenance of the pH value so that the pH increased under the OM treatment after 4 years of cultivation. However, a decreasing pH was observed under the CF treatments due to the heavy application of inorganic N fertilizer, which is common in soil utilized for growing vegetables^33^. If the pH continues to decrease under the CF treatment, we would expect Fe-P to increase proportionally. Conversely, if the pH increases under the OM treatment, we could expect this trend to reverse and P to initially increase in the available forms, after which accumulation might predominantly shift to Ca-Pi. The application of OM also affected the soil bulk density in the 0–20 cm layer, and the value was significantly lower under the OM treatment than under the CF and CK treatments. Total N did not appear to change under any of the fertilizer treatments, which indicated that soil total N remained stable regardless of the fertilization mode.

**Table 6:** Soil fertility parameters under the different treatments after a 4-year experiment.

Treatment	OM g·kg^−1^	pH	BD g·cm^−3^	TN mg·kg^−1^	TP mg·kg^−1^	AN mg·kg^−1^	AP mg·kg^−1^	AK mg·kg^−1^
OM	28.420 ± 4.62aA	7.12 ± 1.71aA	1.03 ± 0.07bB	1836.77 ± 112.43aA	1488.04 ± 87.25aA	78.48 ± 15.26bB	86.04 ± 29.41aA	247.70 ± 28.48aA
CF	20.358 ± 6.34bB	6.75 ± 2.48bA	1.41 ± 0.12aA	1825.26 ± 89.34aA	1392.56 ± 104.38bB	110.60 ± 24.18aA	72.40 ± 25.30bB	213.27 ± 32.17bB
CK	21.375 ± 2.17bB	7.03 ± 1.53aA	1.32 ± 0.10aA	1812.63 ± 65.27aA	1317.70 ± 54.27cC	23.42 ± 3.46cC	19.53 ± 5.43cC	66.58 ± 17.55cC

Note: OM = organic matter; BD = bulk density; TN = total nitrogen; TP = total phosphorus; AN = alkali-hydrolysable nitrogen; AP = available phosphorus; and AK = available potassium. The different lowercase and uppercase letters in the same column indicate a significant difference at the 0.05 and 0.01 levels, respectively.

There was a significant change in nutrient availability under all treatments over time. The available N content decreased significantly in the CK treatment, in which no fertilizer was applied, whereas fertilization increased the available N content under both the OM and CF treatments. However, the concentration of available N under the CF treatment was much higher than that under the OM treatment, which was consistent with the trend of vegetable yields. The result suggested that N was an important limiting factor for vegetable growth, particularly under the OM treatments. The lack of available N is a common problem in organic cultivation in China and results in lower crop yields and more P surplus in the soil because there is a much lower N content than P content in OM, and plant N uptake and N losses are also much greater than P losses in the soil. The available soil K significantly increased under the OM and CF treatments with fertilization and decreased under the CK treatment compared with the values from 2009, which suggested that excessive fertilization could give rise to the accumulation of available K regardless of the fertilization mode.

## Conclusions

The excessive application of P fertilizer resulted in Olsen-P accumulation in the soil, regardless of the use of OM or CF, during the experimental period; however, vegetable yields were significantly lower with OM than with CF, which resulted in more P surplus in the soil under the OM mode. Ca_2_-P and Ca_8_-P were the predominant inorganic forms under OM application, whereas Al-P and Fe-P were greater under CF application. CF application is prohibited in organic farming. To increase vegetable yields, continuous annual application of OM has been regarded as the only effective strategy by farmers; therefore, excessive application of fertilizer is common in vegetable greenhouses in China. In fact, the practice not only has almost no effect on vegetable yields but also results in an increase in soil P levels, which increases the risk for the diffuse pollution of surface waters. Therefore, nutrient management in the greenhouse must be carefully considered when designing high-efficiency cropping systems with P utilization and a low environmental impact in organic farming.
